# Activation of heme-free soluble guanylate cyclase with cinaciguat has beneficial cardiorenal actions when added to furosemide in experimental heart failure

**DOI:** 10.1186/1471-2210-11-S1-P10

**Published:** 2011-08-01

**Authors:** Guido Boerrigter, Lisa C Costello-Boerrigter, Alessandro Cataliotti, Johannes-Peter Stasch, John C Burnett

**Affiliations:** 1 Cardiorenal Research Laboratory, Mayo Clinic, Rochester, MN, USA; 2 Cardiovascular Research, Bayer Schering Pharma AG, Wuppertal, Germany

## Background

Volume overload and sodium retention in congestive heart failure (HF) are usually treated with loop diuretics. However, worsening renal function is frequently observed and is associated with worse outcomes. Soluble guanylyl cyclase (sGC) plays an important role in renal function. Importantly, sGC activation can be impaired in cardiorenal disease states, which can be due to not only decreased bioavailability of nitric oxide but also to heme oxidation or heme loss of sGC, which renders the enzyme insensitive to NO. Cinaciguat is a novel sGC activator that stimulates the heme-free, NO-insensitive form of sGC.

## Hypothesis

Cinaciguat unloads the heart and increases renal blood flow without compromising renal function when added to furosemide in experimental HF.

## Methods

HF was induced in canines (n=13) by tachypacing. On day 11 of pacing, an acute study was performed under general anesthesia. The left ureter was catheterized and the renal artery was equipped with a flow probe. After equilibration a 30-minute baseline clearance (C1) was done. After this, animals were assigned to one of two groups. One group received furosemide (1 mg/kg/h) for 90 minutes, the other group received the same dose of furosemide plus cinaciguat (0.1 followed by 0.3 µg/kg/min for 45 minutes each). Two clearances (C2 and C3) were done from 15-45 and from 60-90 minutes of drug infusion. After a 30-minute washout a post-infusion clearance (C4) was done. *p<0.05 between groups.

## Results

In the furosemide group, mean arterial pressure was not changed during drug administration but decreased during C4. Cardiac output and systemic and pulmonary vascular resistances were unchanged. Right atrial, pulmonary artery, and pulmonary capillary wedge pressures were reduced during C3 and C4. In the furosemide+cinaciguat group, mean arterial pressure* and systemic* and pulmonary* vascular resistances decreased in C2*, C3*, and C4*. The same was true for right atrial*, pulmonary artery* and pulmonary capillary wedge pressures*. Cardiac output increased in C2*, C3*, and C4*. Furosemide alone did not change renal blood flow, whereas furosemide+cinaciguat increased it in C2, C3*, and C4. Urine flow and urinary sodium excretion increased in both groups during C3 and C4 with no differences between groups. Glomerular filtration rate increased with furosemide alone during drug infusion, while this tended to be the case in the furosemide+cinaciguat group, with no differences between groups (p>0.4).

## Conclusion

Cinaciguat when added to furosemide reduces cardiac preload and afterload and increases renal blood flow when compared to furosemide alone. Importantly, GFR and sodium excretion were not negatively affected by cinaciguat, despite the reduction in mean arterial pressure. It remains to be established whether these promising findings can translate into improved renal outcomes in patients with HF.

